# Supplementary material for: Climate-driven succession in marine microbiome biodiversity and biogeochemical function
Source: Nat Commun. 2025 Apr 25;16:3926. doi: 10.1038/s41467-025-59382-1 (PMC12032349; doi:10.1038/s41467-025-59382-1)
Supplement: Supplementary file 1 — Supplementary Information [file 41467_2025_59382_MOESM1_ESM.pdf]

Title: Climate-driven succession in marine microbiome biodiversity and biogeochemical function

Authors: Alyse A. Larkin[1], Melissa L. Brock[2], Adam J. Fagan[1], Allison R. Moreno[2,3], Skylar D. Gerace[1], Lauren E. Lees[2], Stacy A. Suarez[2], Emiley A. Eloe-Fadrosh[4], and Adam Martiny[1,2]

[1]Department of Earth System Science, University of California, Irvine, CA, USA
[2]Department of Ecology and Evolutionary Biology, University of California, Irvine, CA, USA
[3]Ocean Sciences Department, University of California, Santa Cruz, CA, USA
[4]US Department of Energy Joint Genome Institute, Lawrence Berkeley National Laboratory, Berkeley, CA, USA

Supplementary Information

Additional Sequencing Methods

31 metagenomic libraries were sequenced from samples collected in 2021. A Nextera DNA Flex Library Prep Kit (Illumina) was used to prepare sequence libraries using a low-volume methodology based on (Adams *et al.*, 2020). Tagmentation was performed with 25-500 ng of DNA in 5 µL reactions (1 µL bead-linked transposons, 1 µL tagmentation buffer, final volumes adjusted with nuclease-free water) incubated at 55°C for 15 m with a 100°C heated lid. A total of 1 µL tagment stop buffer was immediately added to each library then incubated at 37°C for 15 m with a 100°C heated lid. Libraries were placed on a 96-well magnetic plate until solutions were clear. Supernatant was discarded and bead-linked transposons were washed with a tagment wash buffer (20 µL) three times. Next, the bead-linked transposons were combined with PCR mix (2.75 µL nuclease-free water, 0.5 µL KAPA-PCR-F primer (AAT GAT ACG GCG ACC ACC G*A; 10 µM), 0.5 µL KAPA-PCR-R primer (CAA GCA GAA GAC GGC ATA CG*A; 10 µM), 2.5 µL custom Nextera DNA-style 8 bp unique dual index barcodes, and 6.25 µL KAPA KiFi HotStart ReadyMix (2X)). The I7 index sequence is 5′-CAA GCA GAA GAC GGC ATA CGA GAT [NNN NNN NN]G TCT CGT GGG CTC GG-3′, and the I5 index sequence is 5′-AAT GAT ACG GCG ACC ACC GAG ATC TAC AC[N NNN NNN N]TC GTC GGC AGC GTC-3′. PCR barcoding was performed using the following reaction: 72°C for 3 m, 98°C for 3 m, followed by 12 cycles of: 98°C for 45 s, 62°C for 30 s, 72°C for 2 m, and a final extension step at 72°C for 1 m. After PCR, libraries were placed on a magnetic stand until the supernatant was clear. The supernatant was then quantified using a Qubit dsDNA HS Assay kit and a Synergy 2 Microplate Reader and equimolar concentrations of each library were pooled.

A two-step magnetic bead selection protocol was used for size selection. First, 45 µL of pooled libraries were incubated at room temperature with 56 µL of sample purification beads and 29 µL of nuclease-free water for 5 m. Second, 120 µL of supernatant from the first reaction was incubated with 12 µL of sample purification beads at room temperature for 5 minutes. The supernatant was removed and sample purification beads were washed two times with 200 µL of 80% ethanol. A total of 32 µL of resuspension buffer was added to the purification beads and incubated in a water bath at 50°C for 5 m to ensure complete elution of the pooled library. Quantification of the final library was performed using a Qubit dsDNA HS Assay and Qubit fluorometer and via KAPA qPCR. A 2100 Bioanalyzer high sensitivity DNA trace was used to determine fragment size distribution. Finally, the pooled library was sequenced (150 bp, paired-end) on one lane of an Illumina NovaSeq 6000 S4 flow cell. Sequencing produced a total of 0.259 Tbp with an average of 8.36 Gbp/sample.

Detailed Metagenomic Assembly and Annotation

Short-read metagenomes were processed following the Joint Genome Institute (JGI) metagenomics workflows (Clum *et al.*, 2021). Raw paired-end sequences were quality

controlled using rqcfilter2 from BBTools (v38.94) (Bushnell, 2018). Specifically, 'bbduk' was used to remove adapters, perform quality trimming by removing reads where quality drops to zero, removing reads that contain 4 or more "N" bases, removing reads that have an average quality score < 3, and removing reads with a minimum length of < 51 bp. Bbduk was also used for artifact removal of homopolymer stretches of 5 G's or more at the ends of reads. Bbmap was used to remove reads that matched at 93% identity to host and common microbial contaminant sequences. Read error correction was performed using 'bbcms' from BBTools (v38.94) with a minimum count of 2 and a high-count fraction of 0.6. Corrected reads were then assembled using metaSPAdes (v3.15.0) (Nurk *et al.*, 2017) with the 'metagenome' flag and kmer sizes of 33, 55, 77, 99, and 127. Contigs smaller than 200 bp were discarded. Assembled reads were mapped back to the contigs to obtain coverage information using 'bbmap' from BBTools (v38.94) with 'interleaved' as true, 'ambiguous' as random, and the 'covstats' option specifying a contig coverage file. The assembled reads were structurally annotated using tRNAscan-SE (v2.0) (Chan *et al.*, 2021), RFAM (Kalvari *et al.*, 2021), CRT-CLI (v1.8), Prodigal (v2.6.3) (Hyatt *et al.*, 2010), and GeneMarkS-2 (v1.07) (Lomsadze *et al.*, 2018) as previously described (Clum *et al.*, 2021). Structural annotation results were merged to create a consensus structural annotation, which was then used for functional annotation. On average, 71.99% of quality filtered reads (Minimum: 22.67%, Maximum: 100%) were assembled.

Functional annotations were assigned using multiple protein family databases: KO (Mao *et al.*, 2005), EC (Ryu, Kim and Lee, 2019), COG (Tatusov *et al.*, 2000), TIGRFAM (Haft, Selengut and White, 2003), and Pfam (Finn *et al.*, 2014). With the exception of KEGG Orthology, all other assignments are done using hmmsearch from HMMER 3.1b2 package (Eddy, 2023), with model-specific trusted cutoff for Pfam, noise cutoff for TIGRFAM or with --domE 0.01 cutoff for the rest of the families. KEGG Orthology Terms are assigned using lastal 983 against KEGG Genes v77.1 to assign KO Terms to IMG-NR genes, which is then used to assign KO Terms to the rest of the genes. Protein product names are assigned based on the name of their associated protein families in the order of priority KO term > TIGRFAM > COG > Pfam. Proteins without any of the above-mentioned assignments are annotated as a "hypothetical protein." Phylogenetic assignments for each read were assigned based on the best Last hits of the protein coding genes (CDSs). A consensus phylogenetic assignment for each contig was generated using a majority rule, whereby the lineage at the lowest taxonomic rank to which at least 50% of CDSs on the contig was assigned. JGI's pipeline can be implemented using the National Microbiome Data Collaborative's (NMDC) open-source online platform Empowering the Development of Genomics Expertise (EDGE) (Eloe-Fadrosh *et al.*, 2022).

Supplementary References

Adams, E. *et al.* (2020) Low volume methodology for Nextera DNA Flex library prep kit (96 samples). Available at: https://www.protocols.io/view/low-volume-methodology-for-nextera-dna-flex-librar-be6rjhd6.

Bushnell, B. (2018) BBTools: a suite of fast, multithreaded bioinformatics tools designed for analysis of DNA and RNA sequence data, *Joint Genome Institute*.

Chan, P.P. *et al.* (2021) tRNAscan-SE 2.0: improved detection and functional classification of transfer RNA genes, *Nucleic Acids Research*, **49**(16), 9077–9096.

Clum, A. *et al.* (2021) DOE JGI Metagenome Workflow, *mSystems*, **6**(3),10-1128.

Eddy, S.R. (2023) 'HMMER'. Available at: http://hmmer.org/.

Eloe-Fadrosh, E.A. *et al.* (2022) The National Microbiome Data Collaborative Data Portal: an integrated multi-omics microbiome data resource, *Nucleic Acids Research*, **50**(D1), D828–D836.

Finn, R.D. *et al.* (2014) Pfam: the protein families database, *Nucleic Acids Research*, **42**(D1), D222–D230.

Haft, D.H., Selengut, J.D. and White, O. (2003) The TIGRFAMs database of protein families, *Nucleic Acids Research*, **31**(1), 371–373.
Hyatt, D. *et al.* (2010) Prodigal: prokaryotic gene recognition and translation initiation site identification, *BMC Bioinformatics*, **11**(1), 119.
Kalvari, I. *et al.* (2021) Rfam 14: expanded coverage of metagenomic, viral and microRNA families, *Nucleic Acids Research*, **49**(D1), pp. D192–D200.
Lomsadze, A. *et al.* (2018) GeneMarkS-2: raising standards of accuracy in gene recognition.
Mao, X. *et al.* (2005) Automated genome annotation and pathway identification using the KEGG Orthology (KO) as a controlled vocabulary, *Bioinformatics*, **21**(19), 3787–3793.
Nurk, S. *et al.* (2017) metaSPAdes: a new versatile metagenomic assembler, *Genome Research*, **27**(5), 824–834.
Ryu, J.Y., Kim, H.U. and Lee, S.Y. (2019) Deep learning enables high-quality and high-throughput prediction of enzyme commission numbers, *Proceedings of the National Academy of Sciences*, **116**(28), 13996–14001.
Tatusov, R.L. *et al.* (2000) The COG database: a tool for genome-scale analysis of protein functions and evolution, *Nucleic Acids Research*, **28**(1), 33–36.

Supplementary Figures

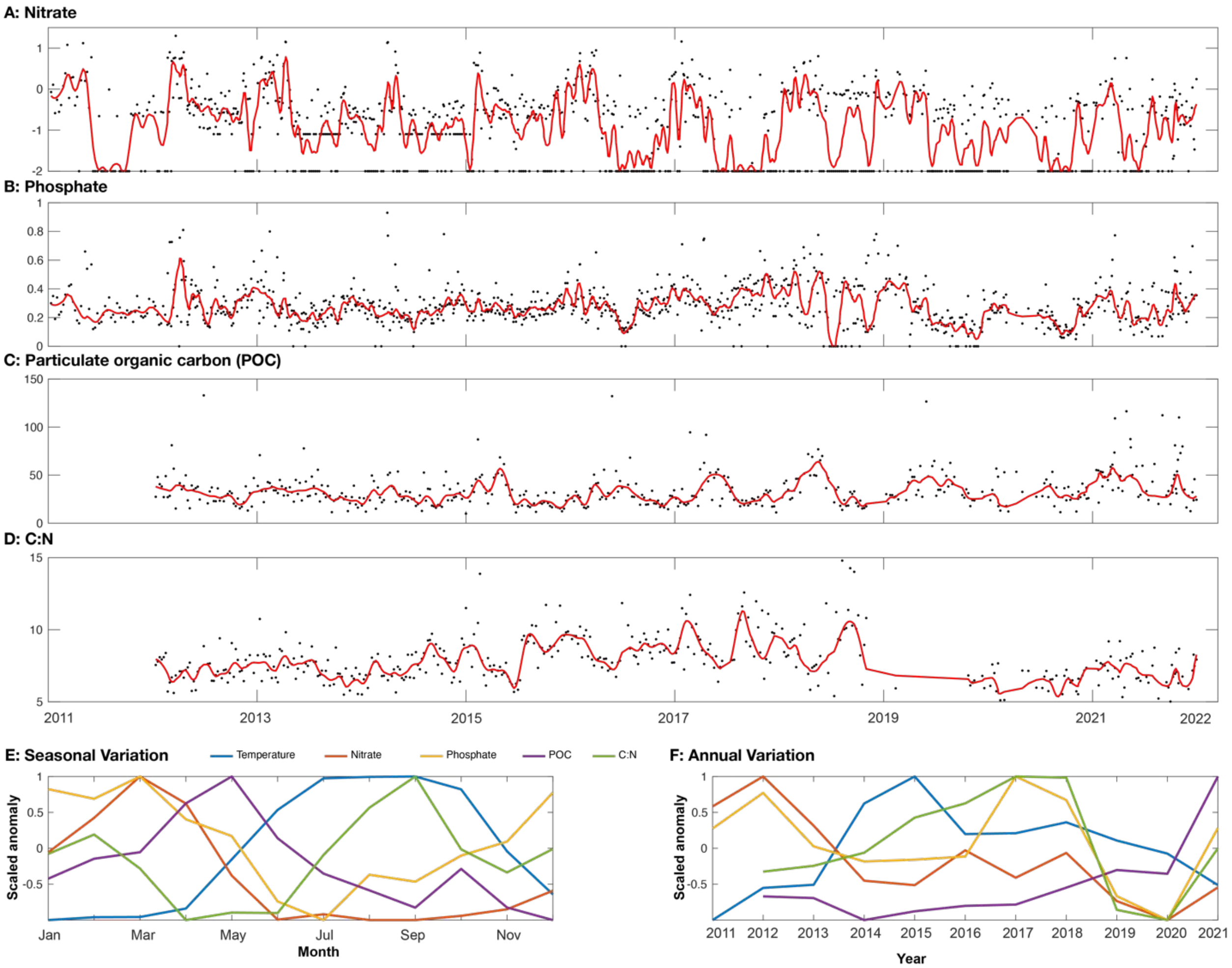

Supplementary Fig. 1. Additional environmental dynamics at MiCRO. A: Nitrate (μM). **B:** Phosphate (μM). **C:** Particulate organic carbon (POC, μM). **D:** Particulate organic carbon-to-nitrogen ratio (C:N, mol:mol). **E:** Monthly anomaly in temperature, nitrate and phosphate, POC, and C:N. **F:** 2011 to 2021 annual anomalies in temperature, nitrate and phosphate, POC, and C:N. Anomalies were scaled from -1 to 1. Red lines represent a moving average using 'Robust Loess' with a window size = 15.

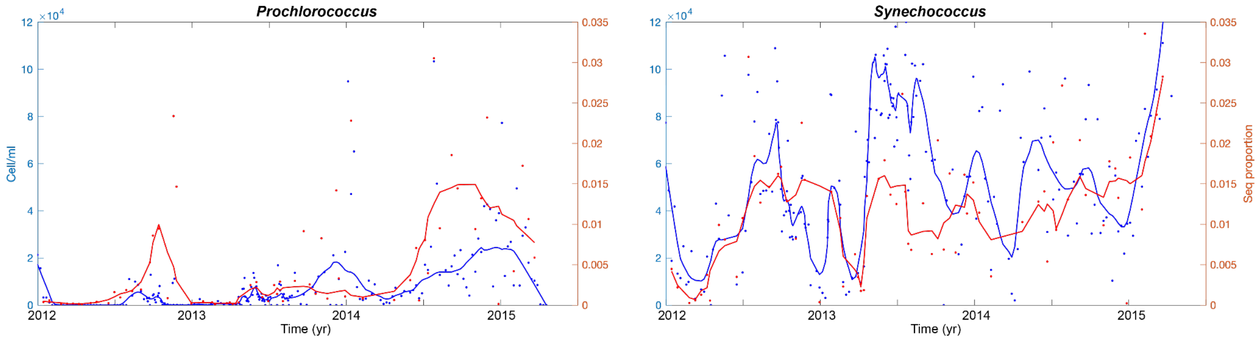

Supplementary Fig. 2. Comparison between abundance measured with flow cytometry and proportion estimated from metagenomics for *Prochlorococcus* and *Synechococcus*. Correlation between absolute FCM counts (blue) and proportion from metagenomics (red) was R_{Pro} = 0.78, p < 1.7E-15 and R_{Syn} = 0.72, p < 2.8E-12. Data underlying the figure are provided in FigShare (see Data Availability).

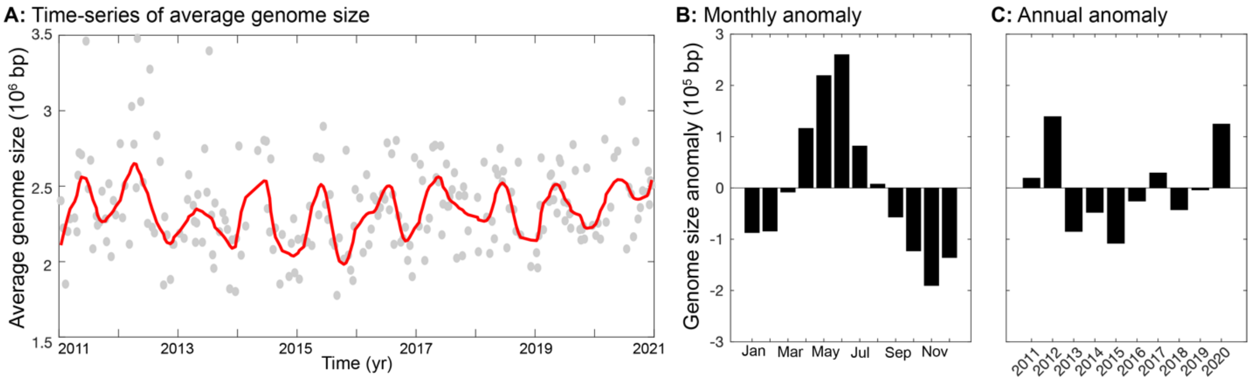

Supplementary Fig. 3. Seasonal and interannual changes in average genome size. A: Time-series of average genome size. Red line represents the moving average calculated using 'Robust Loess' and window size = 20. **B:** Monthly anomaly in genome size. **C:** Annual anomaly in genome size.

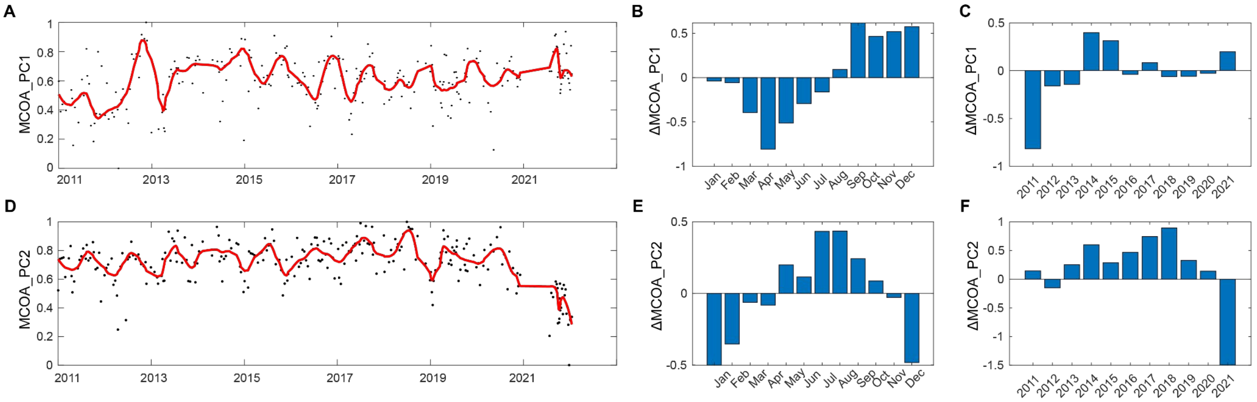

Supplementary Fig. 4. Seasonal and long-term succession in microbial community using Multi-table Co-Inertia Analysis (MCOA). A: Time-series of the first principal component (MCOA_PC1, 35% variance) normalized to 0 to 1. Overlaid is a moving averaged estimated with robust loess. Monthly **(B)** and interannual **(C)** anomaly in MCOA_PC1. **D:** Time-series of the second principal component (MCOA_PC2, 16% variance) normalized to 0 to 1. Overlaid is a moving averaged estimated with robust loess. Monthly **(E)** and interannual **(F)** anomaly in MCOA_PC2. The MCOA analysis includes gene annotations from KEGG, COG, PFam, and TIGRfam as well as taxonomic variation (family level). Data underlying the figure are provided in FigShare (see Data Availability).

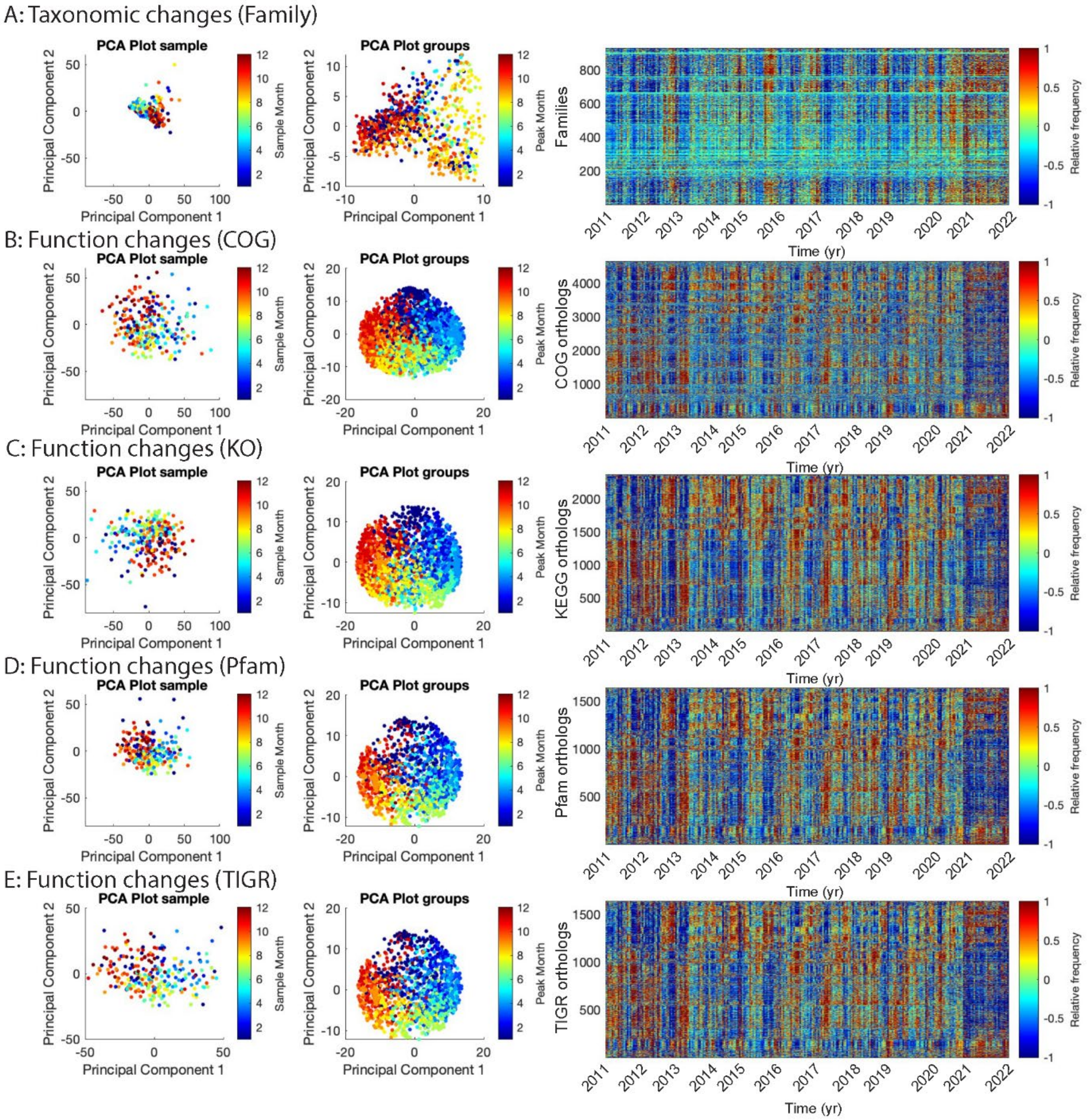

Supplementary Fig. 5. Seasonal and long-term succession in microbial community functional potential across annotation systems. Each row includes the temporal dynamics of community taxonomy or functional potential, where sample similarity was estimated from Z-score normalized relative abundances using PCA (each point represents a sample); the integrated community taxonomic or functional similarity using multi-table co-inertia analysis (MCOA) (each point represents a gene or taxa); and, the time-series of taxa or genes annotated with an ortholog system, where the frequency of each annotation was Z-score normalized across time. Data underlying the figure are provided in FigShare (see Data Availability).

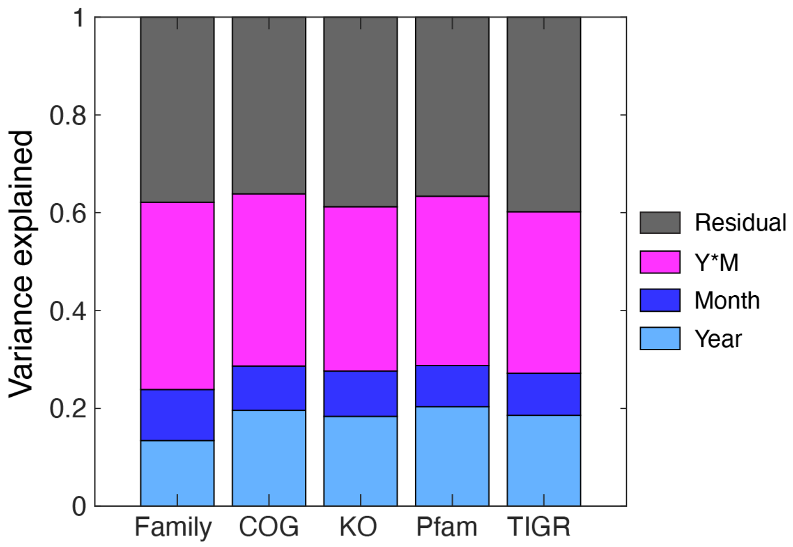

Supplementary Fig. 6. Community compositional variance attributed to interannual and seasonal variation. Compositional variance is estimated using PERMANOVA with sampling 'Year' and 'Month' as factors plus interactions. Composition was estimated using the frequency of taxonomic groups at family level as well as functional genes classified with COG, KEGG Orthologs, Pfam, or TIGR families. The mean compositional variance attributed to interannual plus seasonal changes was 62%. Data underlying the figure are provided in FigShare (see Data Availability).

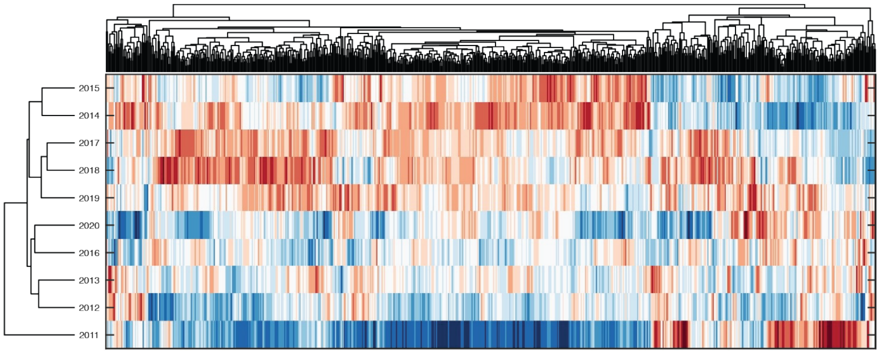

Supplementary Fig. 7. Clustergram of interannual microbiome potential. The annual anomaly for KEGG ortholog was estimated and then grouped using hierarchical clustering analysis with euclidean distances and average linkage. Blue indicates relatively more frequent, and red indicates relatively less frequent genes. Data underlying the figure are provided in FigShare (see Data Availability).

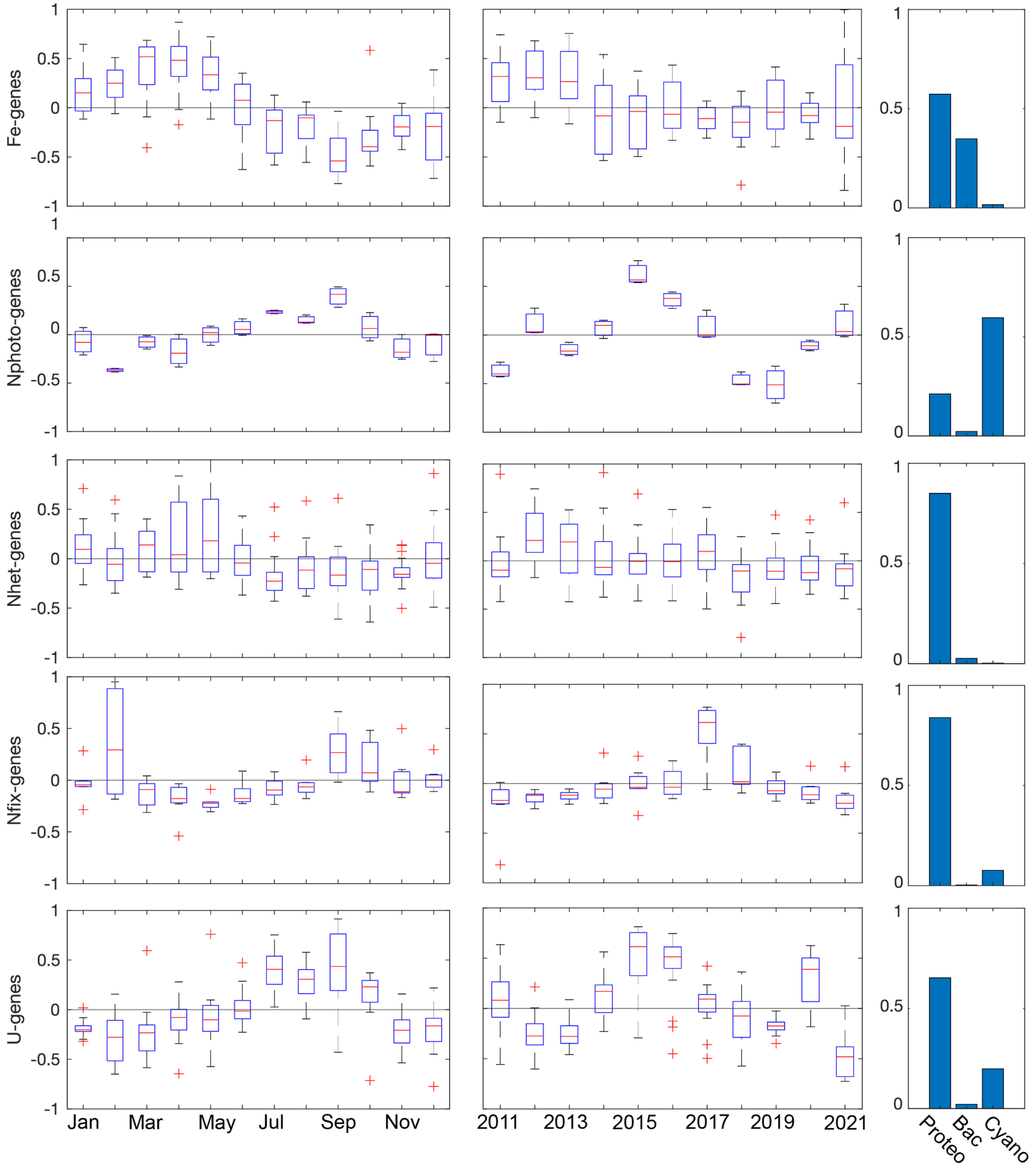

Supplementary Fig. 8. Seasonal and long-term changes of community aggregated nutrient stress traits (Fe and N) and their phylogenetic origin. The genes and associated annotation for each trait are listed in Supplementary Data 2. Box plots represent anomalies of all genes in each functional category. The box consists of the median (center line), 25 and 75 percentile (box), whiskers cover 99.3% of points, and crosses are outliers. Barplots show the proportion of genes annotated as Proteobacteria, Bacteroides, and Cyanobacteria. Data underlying the figure are provided in FigShare (see Data Availability).

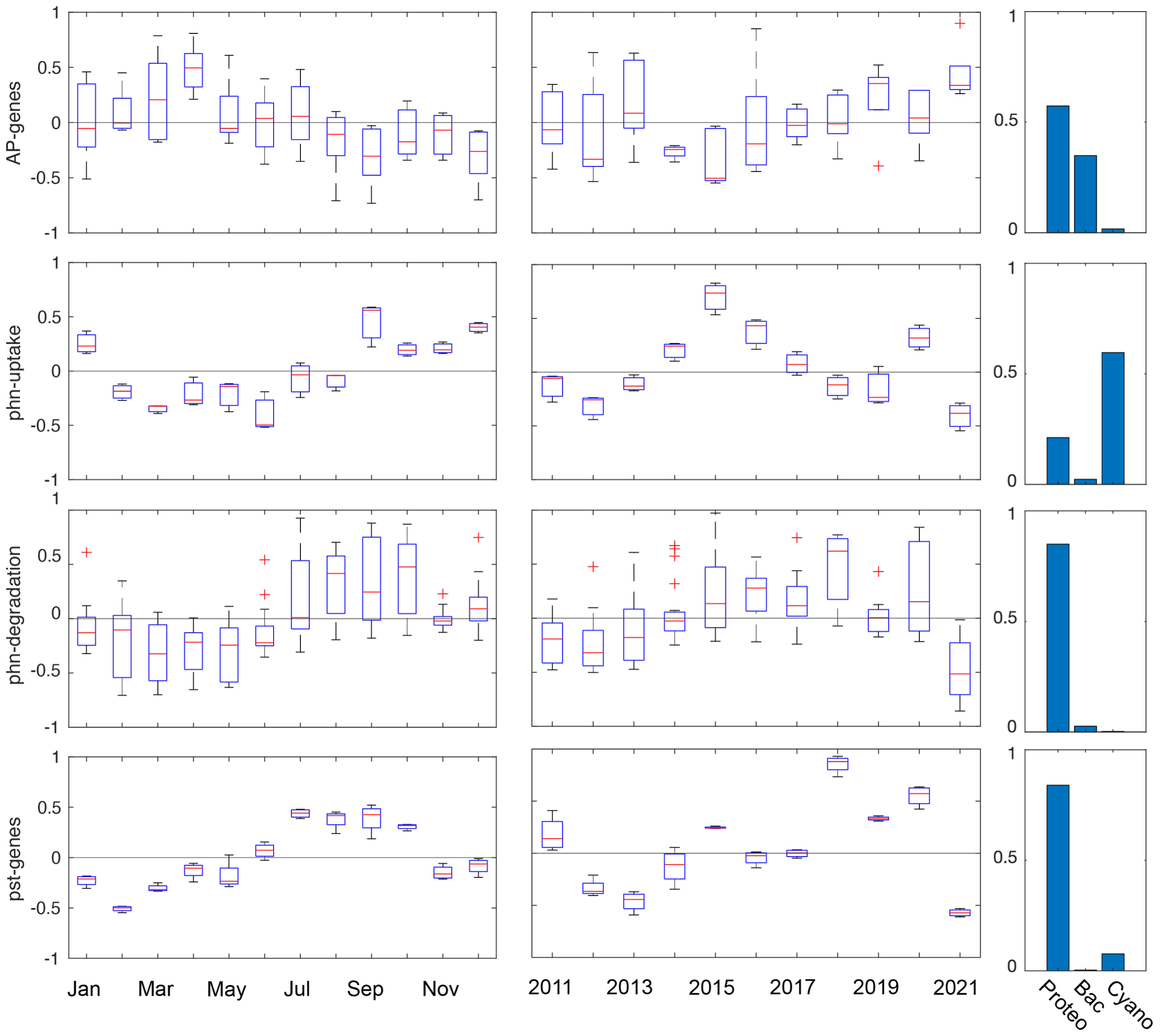

Supplementary Fig. 9. Seasonal and long-term changes of community aggregated nutrient stress traits (P) and their phylogenetic origin. The genes and associated annotation for each trait are listed in Supplementary Data 2. Box plots represent anomalies of all genes in each functional category. The box consists of the median (center line), 25 and 75 percentile (box), whiskers cover 99.3% of points, and crosses are outliers. Barplots show the proportion of genes annotated as Proteobacteria, Bacteroides, and Cyanobacteria. Data underlying the figure are provided in FigShare (see Data Availability).

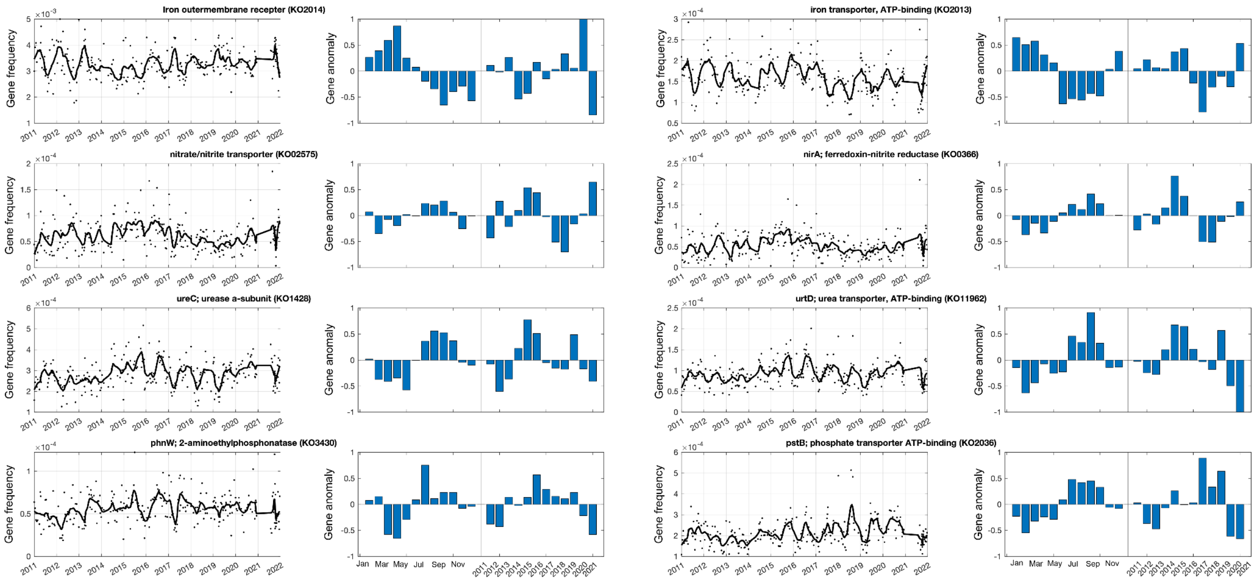

Supplementary Fig. 10. Seasonal and long-term changes of specific nutrient stress genes. Genes include (i) KO2014, "TC.FEV.OM; iron complex outermembrane recepter protein", KO2575, "ABC.FEV.A; iron complex transport system ATP-binding protein [EC:7.2.2.-]", KO2013, "NRT2, narK, nrtP, nasA; MFS transporter, NNP family, nitrate/nitrite transporter", KO0366, "nirA; ferredoxin-nitrite reductase [EC:1.7.7.1]", KO1428, "ureC; urease subunit alpha [EC:3.5.1.5]", KO11962, "urtD; urea transport system ATP-binding protein", KO3430, "phnW; 2-aminoethylphosphonate-pyruvate transaminase [EC:2.6.1.37]", KO2036, "pstB; phosphate transport system ATP-binding protein [EC:7.3.2.1]". Solid black line is a moving average (using lowess) and dots are sample frequencies of genes. The blue bars represent monthly and yearly normalized anomalies. Data underlying the figure are provided in FigShare (see Data Availability).
