# Supplementary material for: Climate-driven succession in marine microbiome biodiversity and biogeochemical function
Source: Nat Commun. 2025 Apr 25;16:3926. doi: 10.1038/s41467-025-59382-1 (PMC12032349; doi:10.1038/s41467-025-59382-1)
Supplement: Supplementary file 2 — Description of Additional Supplementary Files [file 41467_2025_59382_MOESM2_ESM.pdf]

Description of Additional Supplementary Files

File Name: Supplementary Data 1

Description: Summary and metadata for all metagenomic samples.

File Name: Supplementary Data 2

Description: List of genes linked to each biogeochemical category analyzed in this study.
