# Supplementary material for: Climate-driven succession in marine microbiome biodiversity and biogeochemical function
Source: Nat Commun. 2025 Apr 25;16:3926. doi: 10.1038/s41467-025-59382-1 (PMC12032349; doi:10.1038/s41467-025-59382-1)
Supplement: Supplementary file 5 — Reporting Summary [file 41467_2025_59382_MOESM5_ESM.pdf]

Corresponding author(s): Adam Martiny

Last updated by author(s): Feb 5, 2025

Reporting Summary

Nature Portfolio wishes to improve the reproducibility of the work that we publish. This form provides structure for consistency and transparency in reporting. For further information on Nature Portfolio policies, see our Editorial Policies and the Editorial Policy Checklist.

Statistics

For all statistical analyses, confirm that the following items are present in the figure legend, table legend, main text, or Methods section.

n/a	Confirmed	
☐	☒	The exact sample size (*n*) for each experimental group/condition, given as a discrete number and unit of measurement
☐	☒	A statement on whether measurements were taken from distinct samples or whether the same sample was measured repeatedly
☐	☒	The statistical test(s) used AND whether they are one- or two-sided<br>*Only common tests should be described solely by name; describe more complex techniques in the Methods section.*
☐	☒	A description of all covariates tested
☐	☒	A description of any assumptions or corrections, such as tests of normality and adjustment for multiple comparisons
☐	☒	A full description of the statistical parameters including central tendency (e.g. means) or other basic estimates (e.g. regression coefficient) AND variation (e.g. standard deviation) or associated estimates of uncertainty (e.g. confidence intervals)
☐	☒	For null hypothesis testing, the test statistic (e.g. *F*, *t*, *r*) with confidence intervals, effect sizes, degrees of freedom and *P* value noted<br>*Give P values as exact values whenever suitable.*
☒	☐	For Bayesian analysis, information on the choice of priors and Markov chain Monte Carlo settings
☒	☐	For hierarchical and complex designs, identification of the appropriate level for tests and full reporting of outcomes
☐	☒	Estimates of effect sizes (e.g. Cohen's *d*, Pearson's *r*), indicating how they were calculated

Our web collection on statistics for biologists contains articles on many of the points above.

Software and code

Policy information about availability of computer code

Data collection	JGI IMG/M's bioinformatics pipeline was used for generating the data used for our analyses. The pipeline is described in detail in the manuscript.
Data analysis	We used Matlab (v2023a), Python (v3.9.6), and R (v4.3.2) throughout the manuscript. The exact commands and packages are clearly described in the paper. Intermediate data products are also deposited making it easy to recreate and evaluate our analysis

For manuscripts utilizing custom algorithms or software that are central to the research but not yet described in published literature, software must be made available to editors and reviewers. We strongly encourage code deposition in a community repository (e.g. GitHub). See the Nature Portfolio guidelines for submitting code & software for further information.

Data

Policy information about availability of data

All manuscripts must include a data availability statement. This statement should provide the following information, where applicable:

- Accession codes, unique identifiers, or web links for publicly available datasets
- A description of any restrictions on data availability
- For clinical datasets or third party data, please ensure that the statement adheres to our policy

Environmental data can be accessed via the MiCRO BCO-DMO data page (DOI:10.26008/1912/bco-dmo.564351.2)87. Metagenomic sequencing data is available through the Joint Genome Institute Genome Portal (DOI: 10.46936/10.25585/60001365)88 and through the National Center for Biotechnology Information

Sequence Read Archive (BioProject ID: PRJNA624320). For Supplementary Data, including MiCRO metagenome gene and taxon frequencies and analysis, please see FigShare (DOI: 10.6084/m9.figshare.26082091)89. Source Data are provided with this paper.

Research involving human participants, their data, or biological material

Policy information about studies with human participants or human data. See also policy information about sex, gender (identity/presentation), and sexual orientation and race, ethnicity and racism.

Reporting on sex and gender	NA
Reporting on race, ethnicity, or other socially relevant groupings	NA
Population characteristics	NA
Recruitment	NA
Ethics oversight	NA

Note that full information on the approval of the study protocol must also be provided in the manuscript.

Field-specific reporting

Please select the one below that is the best fit for your research. If you are not sure, read the appropriate sections before making your selection.

☐ Life sciences ☐ Behavioural & social sciences ☒ Ecological, evolutionary & environmental sciences

For a reference copy of the document with all sections, see nature.com/documents/nr-reporting-summary-flat.pdf

Ecological, evolutionary & environmental sciences study design

All studies must disclose on these points even when the disclosure is negative.

Study description	Metagenomic analysis of environmental seawater samples
Research sample	One 2 L seawater sample
Sampling strategy	We filter 2 L of seawater onto a 0.22 um filter for each time-point. We use a standard volume. The experiemental design is a time-series with single samples.
Data collection	We used a bucket to collect the water which was subsequently filters. Then DNA extraction and sequencing.
Timing and spatial scale	The exact timing of all the sampling is listed in the paper. We always collected the water from the same spot in Newport Beach. The coordinates for the collection spot is presented. The first sample is collected 2011/01/12 and the last on 2022/01/26
Data exclusions	We used a standard quality cut-off for inclusion of sequences in the analysis. This is standard JGI procedures.
Reproducibility	No replicates were collected
Randomization	We did not do any randomizations
Blinding	NA

Did the study involve field work? ☒ Yes ☐ No

Field work, collection and transport

Field conditions	We drove to the local pier and used a bucket to collect seawater. We did not determine the sampling time based on any environmental conditions.
Location	Newport Pier (33.608°N and 117.928°W)
Access & import/export	It is a local site with no restrictions
Disturbance	It is a random sample so we made no attempts to correct for disturbances

Reporting for specific materials, systems and methods

We require information from authors about some types of materials, experimental systems and methods used in many studies. Here, indicate whether each material, system or method listed is relevant to your study. If you are not sure if a list item applies to your research, read the appropriate section before selecting a response.

Materials & experimental systems

n/a	Involved in the study
☒	☐ Antibodies
☒	☐ Eukaryotic cell lines
☒	☐ Palaeontology and archaeology
☒	☐ Animals and other organisms
☒	☐ Clinical data
☒	☐ Dual use research of concern
☒	☐ Plants

Methods

n/a	Involved in the study
☒	☐ ChIP-seq
☒	☐ Flow cytometry
☒	☐ MRI-based neuroimaging

Plants

Seed stocks	NA
Novel plant genotypes	NA
Authentication	NA
